# Paclitaxel causes degeneration of both central and peripheral axon branches of dorsal root ganglia in mice

**DOI:** 10.1186/s12868-016-0285-4

**Published:** 2016-07-11

**Authors:** Aniqa Tasnim, Zoe Rammelkamp, Amy B. Slusher, Krystyna Wozniak, Barbara S. Slusher, Mohamed H. Farah

**Affiliations:** Neuromuscular Division, Department of Neurology, Johns Hopkins University School of Medicine, The John G. Rangos Sr. Building, Room 239, 855 N. Wolfe Street, Baltimore, MD 21205 USA; Johns Hopkins Drug Discovery Program, Johns Hopkins University School of Medicine, Baltimore, MD USA; Harvard University, Boston, MA USA; University of Maryland Medical School, Baltimore, MD USA

**Keywords:** Neuropathy, Axonal degeneration, Activated macrophages

## Abstract

**Background:**

Peripheral neuropathy is a common and dose-limiting side effect of many cancer chemotherapies. The taxane agents, including paclitaxel (Taxol^®^), are effective chemotherapeutic drugs but cause degeneration of predominantly large myelinated afferent sensory fibers of the peripheral nervous system in humans and animal models. Dorsal root ganglia (DRG) neurons are sensory neurons that have unipolar axons each with two branches: peripheral and central. While taxane agents induce degeneration of peripheral axons, whether they also cause degeneration of central nervous system axons is not clear. Using a mouse model of paclitaxel-induced neuropathy, we investigated the effects of paclitaxel on the central branches of sensory axons.

**Results:**

We observed that in the spinal cords of paclitaxel-intoxicated mice, degenerated axons were present in the dorsal columns, where the central branches of DRG axons ascend rostrally. In the peripheral nerves, degenerated myelinated fibers were present in significantly greater numbers in distal segments than in proximal segments indicating that this model exhibits the distal-to-proximal degeneration pattern generally observed in human peripheral nerve disorders.

**Conclusions:**

We conclude that paclitaxel causes degeneration of both the peripheral and central branches of DRG axons, a finding that has implications for the site and mode of action of chemotherapy agents on the nervous system.

## Background

Chemotherapy-induced peripheral neuropathy (CIPN) is often a factor in limiting cancer therapy with chemotherapeutic drugs, particularly for effective taxane agents such as paclitaxel (Taxol^®^) [1, 2]. CIPN, like most other neuropathies, is characterized by the degeneration of axons, often resulting in sensory or sensory-motor neuropathy in patients. The incidence of CIPN is influenced by a number of factors including dose intensity, cumulative dose, duration of therapy, and co-administration of other neurotoxic chemotherapeutic drugs [3]. Depending on drug dosage and the agent used, symptoms may resolve completely in some cases, but some cases of CIPN are only partly reversible [4]. For such patients, symptoms may persist for a lifetime and cause significant functional impairment and decreased quality of life [5].

Paclitaxel is an effective taxane chemotherapy agent approved for the treatment of breast, ovarian, non-small cell lung carcinomas and Kaposi’s sarcoma. Unfortunately, patients often experience paresthesias, numbness and/or spontaneous pain in a stocking-and-glove distribution, as well as sensory deficits including loss of temperature sensation, decreased vibration perception and sense of position as a result of treatment [6]. Some patients develop neuropathy after only a single administration of the drug, especially when in combination with other chemotherapeutic drugs [4].

In an effort to study the mechanisms responsible for the development of CIPN, numerous animal models have been developed over the years. Rodent models of paclitaxel-induced neuropathy, in particular, show varied degrees of axonal degeneration depending on the dose and treatment schedule. For example, distal axonal degeneration in footpads with no degenerative effects in the sciatic nerve was reported in mice intoxicated with 25 mg/kg body weight administered 3 times per week for 1 week [7]. In rats injected with 2 mg/kg body weight of paclitaxel daily for 6 days, swollen and vacuolated axonal mitochondria were observed with no other reported degeneration [8]. Finally, paclitaxel at a high dose of 60 mg/kg body weight administered 3 times in a single week caused severe degeneration of axons in mice, compared to little axon degeneration seen in mice receiving a low dose of 30 mg/kg 3 times in 1 week [9]. Taken together, previous studies promote the usage of moderately high doses for pathology investigations. Additionally, dose-dependent adverse effects have been observed in the DRG cell bodies of mice. At lower doses (lower than the maximum tolerated dose), dark inclusions as well as clear vacuolations in the cytoplasm of neurons and satellite cells have been observed. At relatively high doses (at the maximum tolerated dose), a proportion of the cytoplasms of DRG neurons appeared much darker than that of normal neurons and a few degenerating neuronal cells were reported [10].

Non-neuronal cells in the nervous system are also affected by chemotherapy agents. Rats injected with paclitaxel have increased numbers of activated microglia and astrocytes in the dorsal horn of the spinal cord, as compared to that of vehicle-treated rats [11, 12]. Activated macrophages appear in both DRG and the sciatic nerves of paclitaxel-intoxicated rats at 18 mg/kg body weight administered 2 times, 3 days apart [11, 12]. In treated rats, Schwann cells in the sciatic nerves and satellite cells of DRG express ATF3, a transcription factor not normally expressed in these cells [11, 12]. While the findings of previous studies have been extensive, they have been mostly limited to peripheral axons, Schwann cells in the sciatic nerve, DRG cell bodies, and non-neuronal cells in the spinal cord; the effect of taxanes on the central branch of DRG sensory axons has not yet been elucidated.

Recently, we developed a paclitaxel-induced neuropathy mouse model that recapitulates aspects of human CIPN. In this model, large and small myelinated axons degenerate and there are pronounced deficits in nerve conduction velocity of caudal and digital nerves [10]. In the present study, we performed morphological analyses of the sciatic nerve and DRG following paclitaxel administration in this model. Symptoms of paclitaxel treatment are shown to manifest in peripheral neuropathy [1, 2], but sensory information is transmitted through the central branch of DRG axons to the central nervous system. Given the degenerative effect of paclitaxel on peripheral sensory axons, we asked whether the central branches of DRG axons are also affected.

## Results

We sought to determine whether the chemotherapy agent paclitaxel causes degeneration of central nervous system axons, particularly those that carry sensory information to higher brain regions. We adapted a well-described mouse model of paclitaxel-induced neuropathy [10], in which mice were treated for 2 weeks and tissue samples were harvested for analysis one day after the end of treatment.

### Axonal degeneration in the spinal cord

**Fig. 1:**
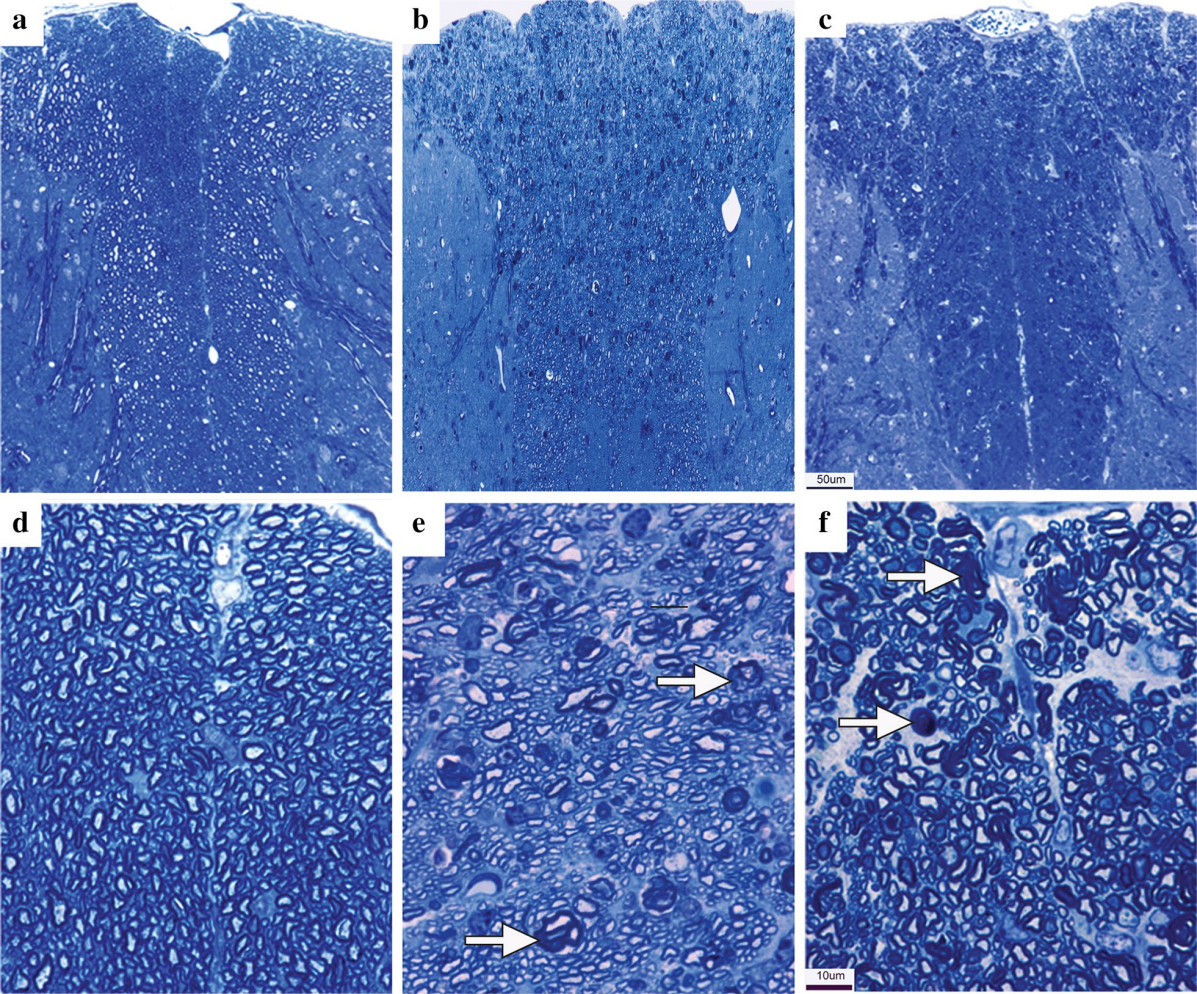
Degeneration of axons in the dorsal column of mice intoxicated with paclitaxel. Images are cross-sectioned semithin (1 µm) plastic sections of spinal cords stained for toluidine *blue*. **a** Control vehicle-treated (cremophor) spinal cord at lumbar level. No degenerated axons are observed. **b** Paclitaxel treated spinal cord. Degenerated myelin profiles are present in dorsal column of thoracic level where ascending central branches axons of DRG neurons reside. **c** Paclitaxel treated spinal cord. Degenerated myelin profiles are present in dorsal column of lumbar level where ascending central branches axons of DRG neurons reside. **d** High magnification of **a** showing no degenerated axons. **e** High magnification of **b** showing degenerated myelin profiles (*arrows*). **f** High magnification of **c** showing degenerated myelin profiles (*arrows*). *Scale bar* in **c** = 50 μm and applies to **a**–**c**. *Scale bar* in **f** = 10 μm and applies to **d**–**f**

**Fig. 2:**
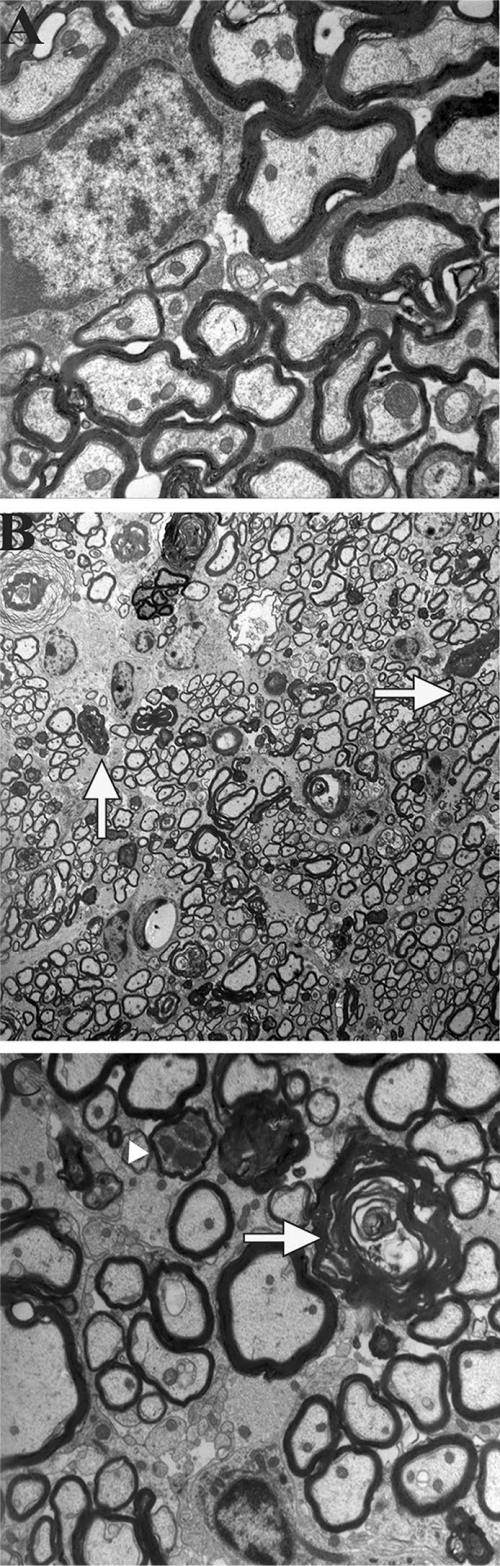
Electron microscope images of degenerated of axons in the dorsal column. **A** EM image showing healthy and no degenerated axons (*arrows*) and myelin ovoids in the ventral horn of lumbar region of mice intoxicated with paclitaxel. **B** Low magnification EM image showing degenerated axons (*arrows*) and myelin ovoids in the dorsal column of lumbar region. **C** High magnification EM images of lumbar level showing degenerated axons at various stages of degeneration. *Arrowhead points* to degenerated axoplasm filled with organelles. *Arrow points* to degenerated myelin profile that has already collapsed following degeneration of axoplasm

Each DRG neuron has a unipolar axon with two branches: peripheral and central. Given the degenerative effect of paclitaxel on peripheral sensory axons, we asked whether the central branches of DRG axons are also affected. We examined representative sections of spinal cords at all levels, and degeneration was observed at all representative levels. In semithin 1 μm plastic sections, degenerated myelin profiles were present in the dorsal column, where central branches of DRG axons ascend rostrally (Fig. 1a–f). Within the spinal cord, degenerated myelin profiles, the footprint of degenerated myelinated axons, were restricted to the dorsal column. None were present in the ventral horn or lateral columns (Fig. 2A), indicating that the toxic effects of paclitaxel were restricted to ascending sensory axons (Fig. 1a–f). Further, the pattern of degeneration in the dorsal column spanned medial and lateral areas of the dorsal column.

As degenerated myelin profiles are not cleared from and persist for years in the central nervous system [13, 14], we examined spinal cords 2 months after paclitaxel treatment, and found degenerated myelin profiles in the dorsal column (data not shown). Our findings suggest that chemotherapy agents can cause degeneration of sensory axons in the central nervous system in addition to the known degeneration of peripheral axons.

Next, we examined the ultra-structural morphology of degenerated axons in the spinal cords of mice intoxicated with paclitaxel under the electron microscope. We observed axons at various stages of degeneration. Axoplasms filled with organelles and disintegrated amorphous granular structures were present with intact myelin (Fig. 2B, C). Myelin ovoid profiles were also present, some with folded and others with unwounded myelin. Degenerated axons were distributed throughout the entirety of the dorsal column, from the dorsal region towards the medial central canal, indicating that both long axons and short ones were affected by paclitaxel toxicity. Taken together, these data suggest that paclitaxel causes degeneration of ascending sensory axons in the CNS.

### Axonal degeneration in the sciatic nerve

**Fig. 3:**
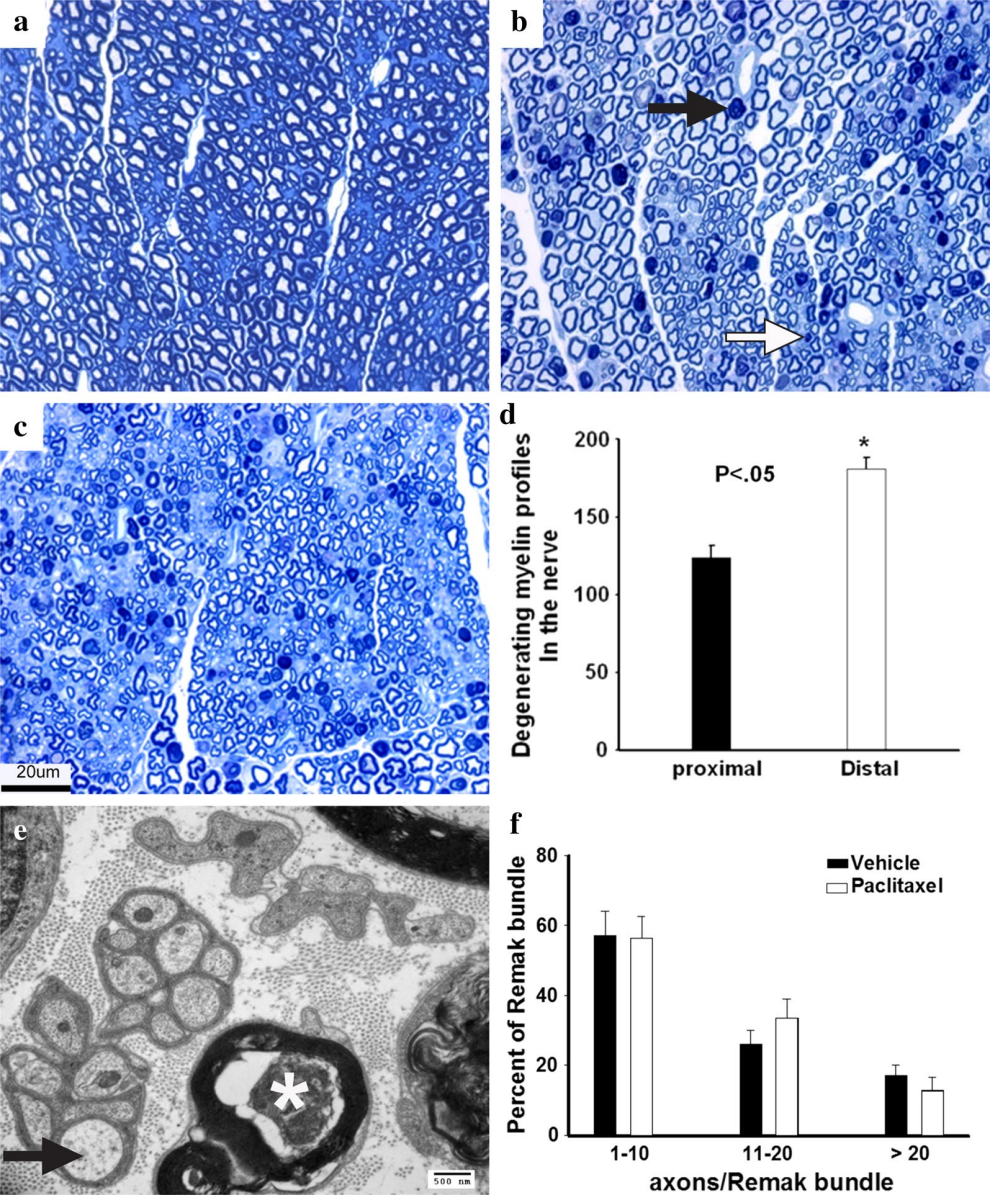
Paclitaxel causes distal to proximal axonal degeneration in mice. Mice were treated with i.v. 30 mg/kg paclitaxel 3 times a week for 2 weeks. **a**–**c** are cross-sectioned semithin (1 µm) plastic sections stained for toluidine *blue*. **a** Vehicle-treated (cremophor) mice at the mid-thigh level of sciatic nerve. No degenerated axons are observed. **b** Paclitaxel-treated mice at the mid-thigh level of sciatic nerve. Degenerated myelin profiles were present across the nerve crossed section. *Arrows point* to large (*black arrow*) and small (*white arrow*) degenerated myelinated axons. **c** Paclitaxel-treated mice at 7–8 mm distal to the sciatic nerve segment in **a**. Greater numbers of degenerated myelin profiles are present across the distal segment of the nerve, indicating distal to proximal degeneration pattern. **d** Quantification of degenerating myelinated fibers in the whole cross-sectional area of nerve segments. N = 3 per nerve segment. Values are mean ± SEM. **e** Electron micrograph of distal nerve intoxicated with paclitaxel showing occasional swollen unmyelinated axon (*arrow*) and degeneration of myelinated axon (*). **f** Quantification of Remak bundles. N = 3 mice per treatment. There was no statistical difference between vehicle- and paclitaxel-treated nerves. *Scale bar* in **c** = 20 μm and applies to **a**–**c**. *Scale bar* in **e** = 500 nm

CIPN is thought to affect distal axons first as symptoms initially appear at distal sites [1, 2]. We examined whether our model exhibits a distal-to-proximal degeneration pattern by counting degenerated axons in the sciatic nerves of paclitaxel-intoxicated mice at two segments, distal and proximal. Degenerated myelin profiles were present all over the cross-sectional areas of the nerves at both proximal (Fig. 3b) and distal (Fig. 3c) segments of paclitaxel-intoxicated nerves. On average, 4–5 % of fibers examined were degenerated. However, greater numbers of degenerated fibers were present in the distal segments as compared to that in the proximal segments (Fig. 3d), indicating that this model of paclitaxel-induced neuropathy exhibits the well-described patterns of distal-to-proximal degeneration seen in peripheral neuropathies [13, 15]. Degenerated axons appeared to be of large- and medium-sized fiber classes (Fig. 3c, d). In intoxicated nerves examined under the electron microscope, very few swollen unmyelinated axons were present, and the vast majority of unmyelinated axons appeared normal (Fig. 3e, f), consistent with observations that in humans, paclitaxel has minimal adverse effects on unmyelinated axons [1, 2].

**Fig. 4:**
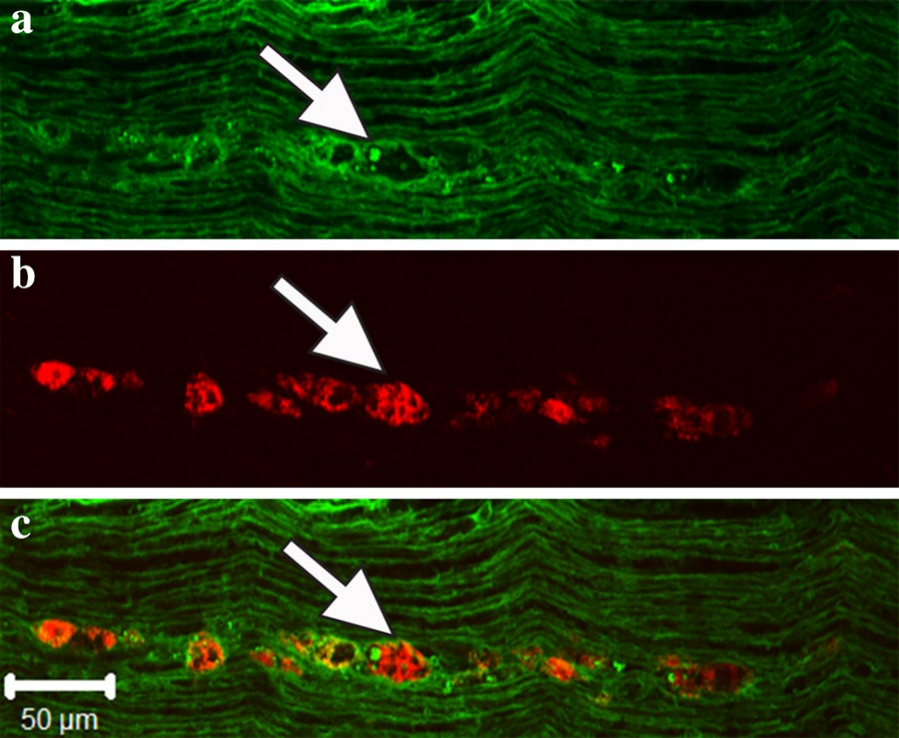
Macrophages infiltration along degenerated axons in nerve intoxicated with paclitaxel. Longitudinal frozen sectioned nerve segment stained for CD68 and MBP. **a** MBP staining of nerve segment intoxicated with paclitaxel. The vast majority of fibers appear normal with the exception of a single fiber that show degenerated myelin (*arrow*). **b** CD68 staining. Macrophages (CD68-positive cells) infiltrate along a degenerating fiber, while not associating with other normal fibers (*arrow*). **c** Merged image of **a** and **b**. *Scale bar* 50 μm

Macrophages infiltrate sciatic nerves in animals intoxicated with paclitaxel [11, 12]. In peripheral neuropathies, macrophages generally associate with degenerated fibers by invading basal lamina and ingesting axonal and myelin debris [13, 16]. We asked whether paclitaxel induced a similar pattern of macrophage invasion in intoxicated nerves. In longitudinal sections, we stained for myelin basic protein (MBP) to identify both healthy and degenerated, fragmented myelinated axons in conjunction with CD68, a lysosomal protein present in activated and phagocytic macrophages [17]. We found macrophages infiltrating degenerating fibers in the midst of normal-looking fibers (Fig. 4).

**Fig. 5:**
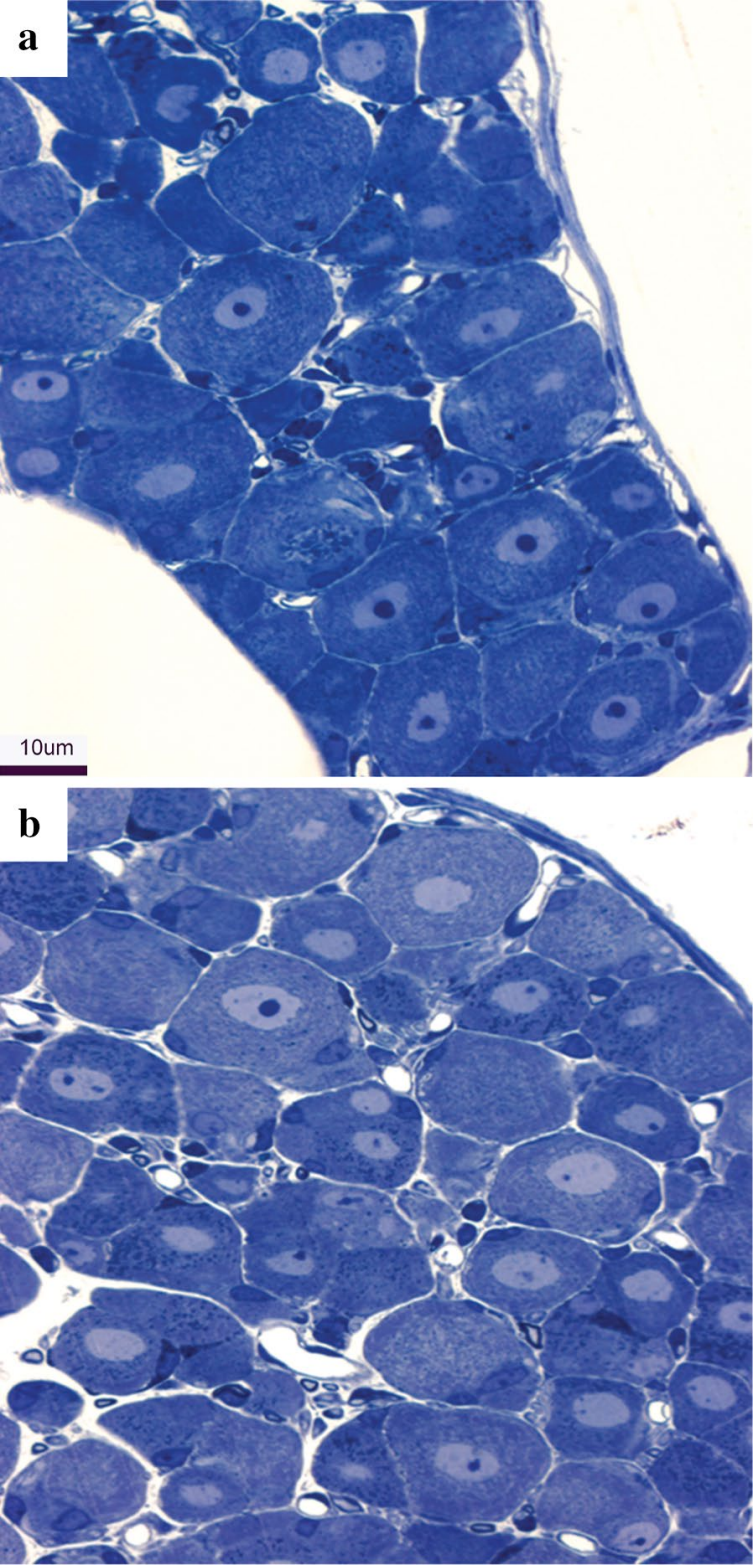
DRG cell bodies after paclitaxel intoxication. **a** Control DRG. **b** Paclitaxel intoxicated DRG. Minor alternations are seen such as small nucleoli in the large neurons of the paclitaxel-treated mice. *Scale bar* in **b** = 10 μm and applies to **a**, **b**

We next examined DRG for signs of neuronal cell body degeneration. We did not observe massive degeneration of neuronal cell bodies (Fig. 5), nor did we observe pyknotic nuclei or fragmented neuronal cells. Instead, we observed minor alterations such as small nucleoli in the large neurons of the paclitaxel-treated mice, supporting the notion that the predominant adverse action of paclitaxel is likely to be on axons, though we cannot exclude contributions from alterations in DRG.

## Discussion

We show that paclitaxel can induce degeneration of the central axons of DRG neurons, building on previously described degenerative effects of taxanes on sensory neurons and axons. Previous work demonstrated that paclitaxel causes peripheral neuropathy that manifests as degeneration of afferent sensory axons [10]. The degree and reversibility of chemotherapy-induced peripheral neuropathy is dependent on the dose and treatment schedule of the chemotherapy agent; recovery is likely due to repair and regenerative capacities of peripheral sensory nerves, resulting in the restoration of some function [1, 2]. However, central axons have limited capacity to repair and regenerate and any functional loss is unlikely to recover [18]. Our work on the mouse model of CIPN, therefore, highlights the critical need for novel approaches of central axonal protection from chemotherapy agents, as opposed to the development of regenerative strategies for peripheral axons only.

DRG neurons have a unique feature of possessing both central and peripheral axonal branches; thus, once inside a neuron, paclitaxel in sufficient doses would be expected to affect microtubules in the cell body and in both axonal branches. Our data suggest that this prediction holds in mouse models. The observation that degeneration is restricted to the dorsal column where DRG axons reside in the spinal cord suggests that paclitaxel does not affect spinal cord axons uniformly and probably does not directly enter the central nervous system in sufficient amounts to cause a uniform toxicity. Indeed, paclitaxel has been shown in relatively low concentrations in the spinal cord compared to sciatic nerve and DRG in mice [2, 19]. Therefore, it is likely that paclitaxel can either be transported from the periphery or directly affect DRG cell bodies to cause degeneration of axons in the dorsal column. Our data cannot distinguish between these two routes, but support the notion that paclitaxel can exert degenerative effects on sensory axons irrespective of whether the axons are in the central or peripheral nervous systems.

A potential explanation for the degeneration of central DRG axons is that at the dose used in this study, paclitaxel may cause massive death of DRG neurons. However, our data argues against this notion as we did not observe degenerating neuronal cells bodies in the DRG of mice intoxicated with paclitaxel (Fig. 5). Other studies also report the absence of massive degeneration of DRG neurons following high dose intoxication [10]. Therefore, in the paclitaxel-induced neuropathy mouse model examined, the degenerative effects of paclitaxel are exerted in part on central sensory axon branches. Importantly, the dose used in this study, 30 mg/kg 3 times a week for 2 weeks, is equivalent to a mg/m^2^ human dose. Paclitaxel is dosed in human at 175–300 mg/m^2^ every 2–3 weeks; thus, the high dose investigated in this study remained within the range that is administered in human. In future studies, it would be interesting to test if similar effects are observed at different doses of paclitaxel intoxication.

In mouse dorsal column and likely in that of human, ascending mechanosensory axons are segregated from axons that transmit proprioceptive information in a medial to lateral pattern [20]. Our data suggest that both types of afferent axons degenerate as we observed degenerated axons in both medial and lateral regions of the dorsal column, and at all levels of the spinal cord of paclitaxel-intoxicated mice. Our findings are consistent with observed effects of taxanes on both mechanoreception and proprioception in human cases [6].

Paclitaxel binds to microtubules and stabilizes them, which interferes with normal cellular function including cell division and microtubule-dependent intra-cellular and axonal transport [4, 6]. Through the aggregation of intracellular microtubules, taxanes affect both the soma of sensory neurons as well as axons [6]. Microtubule-dependent transport is critical for axonal function and health, both in the central and peripheral nervous systems; as such, disruption of axonal transport is implicated in various central nervous system disorders and peripheral neuropathies [21]. Agents such as paclitaxel that disrupt microtubule-dependent transport would be expected to affect both central and peripheral axons given that the agents have access to axons, either directly or via DRG cell bodies.

Involvement of non-neuronal cells in peripheral neuropathy caused by paclitaxel in rodent models has been previously documented [11, 12]. Whether the adverse effect on non-neuroral cells within the nervous system is a primary contributor or a secondary response to axonal injury, however, is an open question. Our data suggest that activated macrophages in paclitaxel-treated nerves are in response to degenerated axons and therefore, at least one cell type, macrophages, mediates secondary effects to the insult of paclitaxel on axons. Foamy phagocytic macrophages were associated with degenerating fibers, but not intact myelinated axons. This pattern of macrophage infiltration has been described in other human peripheral neuropathies, including axonal neuropathies [13]. Therefore, macrophages in paclitaxel-treated nerves might be clearing debris of degenerated axons and myelin, thus allowing for regeneration and recovery over time, and facilitating the resolution of peripheral neuropathy from taxanes in some cases.

Though our data is restricted to the mouse model of CIPN, the results have implications for patients undergoing aggressive chemotherapy treatment. In a few cases, degeneration of central axons has been reported in patients treated with chemotherapy agents. For example, degeneration of the dorsal column of the spinal cord has been described in a patient treated with bortezomib, a proteasome inhibitor that is a very effective chemotherapy agent for multiple myeloma [22]. A limiting factor in identifying cases presenting with dorsal column degeneration has been the requirement for autopsy tissue. However, with the availability of MRI imaging capable of detecting damage to the spinal cord [23], it is now possible to ascertain degeneration in the spinal cords of patients undergoing aggressive treatment. The data in our mouse model of CIPN argue for such examination.

## Conclusions

The data from our mouse model of CIPN suggest that in addition to exerting degenerative effects on peripheral axons, the chemotherapeutic agent paclitaxel also causes degeneration of the central branches of dorsal root ganglia (DRG) ascending in the dorsal spinal cord. These findings contribute to the understanding of the site and mode of action of paclitaxel on the nervous system, and highlight the need to develop novel protective strategies for central axons during aggressive chemotherapy treatment.

## Methods

### Animals

A total of 22 3–4 month old mice on C57Bl/6 background, both male and female, were used. The animal care procedures and experimental protocols were approved by the Animal Care and Use Committee of the Johns Hopkins University School of Medicine.

### Paclitaxel administration

Paclitaxel (LC laboratories) was dissolved in 100 % ethanol. Equal volumes of paclitaxel solution and cremophor (Sigma-Aldrich) were vigorously vortexed for 10 min. Ice-cold saline (80 % of the final volume) was added to freshly made paclitaxel/cremophor solution immediately before injection. 30 mg/kg paclitaxel in this solution was injected intravenously via tail vein at 3 times a week for 2 weeks. Survival time after treatment was 1 day.

### Tissue processing

Mice were deeply anesthetized with 10 % chloral hydrate and killed by transcardial perfusion with 1× PBS followed by 4 % paraformaldehyde in 1× PBS. Tissues were post-fixed in the same fixative overnight. Sciatic nerves were cryoprotected in 30 % sucrose in 1× PBS, and sectioned at 20 μm. Sciatic nerves were sectioned longitudinally using a Leica cryostat (model CM3050S; Leica Microsystems). For semithin sections, nerves, DRG, and spinal cords were postfixed in 4 % paraformaldehyde/2 % glutaraldehyde followed by OsO_4_ and embedded in Epon. Cross sections, at 1 μm thickness, were stained for toluidine blue and examined under light microscopy. For ultra-structural analyses, spinal cord and nerves were processed as described previously [24]. Briefly, 70 nm thin sections were obtained and stained for citrate/uranyl acetate. Electron micrographs were acquired using Zeiss Libra transmission electron microscope.

### Quantification

Counts of degenerated axons across sectional areas of the entire nerves were quantified. Images taken at 60× oil-immersion lens were tiled to obtain montages that cover the entire nerves. Degenerated myelin profiles were counted for each nerve at mid-thigh level and distal segments were taken at 7–8 mm away, and N = 3 for each segment examined. For unmyelinated axon counts, 100–110 Remak bundles were imaged by EM from each nerve. The number of axons per Remak bundle was counted.

### Staining protocol

For immunocytochemistry, nonspecific antibody binding was blocked by 5 % goat serum (Jackson labs)/0.3 % Triton (Sigma-Aldrich) in 1× PBS overnight at room temperature. Sections were then incubated with primary antibodies MBP (Millipore) and CD68 (AbD Serotec) overnight at 4 °C. After 3× PBS washes, sections were incubated with appropriate anti-rabbit and -rat secondary antibodies conjugated with Alexa Fluor 488 and 546 (Life technologies) at a 1:2000 dilution for two hours at room temperature. Samples were washed three times in PBS, mounted (Prolong Antifade kit; Life technologies), and cover-slipped. Stained sections were examined under confocal microscope (LSM500, Zeiss).

### Statistical analysis

All statistical analyses were performed using the Student’s t test. SigmaPlot software was used to perform the analyses and any value of p < 0.05 was scored as statistically significant. Graphed data are presented as mean ± SEM.

## Abbreviations

PBS: phosphate buffer saline
EM: electron microscope
